# 40 Evaluation of a Multidisciplinary Approach to Pressure Injury Prevention Among Patients in a Burn Center

**DOI:** 10.1093/jbcr/irac012.043

**Published:** 2022-03-23

**Authors:** Stacey Richerbach, Tiffany Hockenberry, Karen Richey, Kevin N Foster

**Affiliations:** Arizona Burn Center, Phoenix, Arizona; Arizona Burn Center Valleywise Health, Phoenix, Arizona; Arizona Burn Center Valleywise Health, Phoenix, Arizona; The Arizona Burn Center Valleywise Health, Phoenix, Arizona

## Abstract

**Introduction:**

Compromised skin integrity in tandem with the prolonged length of stay necessitated by patients in a burn center has the capacity to result in an increased incidence of hospital acquired pressure ulcers (HAPUs). A HAPU increases the cost of a hospital stay by approximately $43,000. Many interventions exist to reduce their occurrence; however, interventions have been largely compartmentalized by discipline. One burn center’s dramatic increase in HAPUs from 2018 to 2019 led to further evaluation of prevention efforts. Upon detecting a 117% increase of HAPUs and a threefold increase in cost for specialty bed rental, a multidisciplinary approach to pressure injury prevention was employed. The frequency of multidisciplinary patient rounds was increased from once to twice daily, and involved discussions regarding functional status, mobility, and nutrition. The practice of ordering specialty beds was modified to require three-part approval by the Medical Director of Burn Services, Nursing Director, and a burn therapy supervisor. The purpose of this QI project was to examine the implementation of the multidisciplinary approach to pressure injury prevention among patients in a burn center and to identify trends in HAPUs and specialty bed expenses.

**Methods:**

This was a retrospective review of patients admitted to a Burn Center between January 1, 2018 and June 30, 2021. Preexisting pressure ulcers were identified upon admission and were not regarded as HAPUs. Patients were grouped by year of their admission date.

**Results:**

A total of 941 patients were admitted for inpatient care in 2020, six HAPUs (1.35%) were identified for this population. As compared to the prior year, this revealed a 53% decrease in incidence of HAPUs. The specialty bed rental cost from 2019 to 2020 was reduced by $110,181.76 (68%).

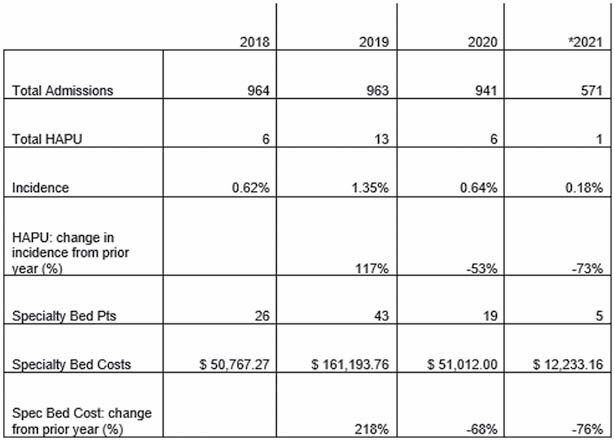

*Data provided for 2021 is from 01/01/2021 to 06/30/2021

**Conclusions:**

Following implementation of a multidisciplinary approach to pressure injury prevention, we were able to reduce the incidence of HAPUs and dramatically reduce the expense associated with specialty bed rental. This supports an effort to identify additional practices which may increase quality while decreasing cost.

